# U-IMD: the first Unified European registry for inherited metabolic diseases

**DOI:** 10.1186/s13023-021-01726-3

**Published:** 2021-02-18

**Authors:** Thomas Opladen, Florian Gleich, Viktor Kozich, Maurizio Scarpa, Diego Martinelli, Franz Schaefer, Kathrin Jeltsch, Natalia Juliá-Palacios, Ángels García-Cazorla, Carlo Dionisi-Vici, Stefan Kölker

**Affiliations:** 1 Division of Neuropediatrics and Metabolic Medicine, Department of General Pediatrics, Centre for Child and Adolescent Medicine, Im Neuenheimer Feld 430, 69120 Heidelberg, Germany; 2 Department of Pediatrics and Inherited Metabolic Disorders, Charles University - First Faculty of Medicine and General University Hospital, Prague, Czech Republic; 3 Regional Coordinating Center for Rare Diseases, Udine University Hospital, Udine, Italy; 4 U.O.C. di Patologia Metabolica, Dipartimento di Medicina Pediatrica, IRCCS Ospedale Pediatrico Bambino Gesù, Rome, Italy; 5 Inborn Errors of Metabolism Unit, Neurology Department, Institut de Recerca Sant Joan de Déu, and CIBERER-ISCIII, Barcelona, Spain; 6 Division of Pediatric Nephrology, Department of General Pediatrics, Centre for Child and Adolescent Medicine, Heidelberg, Germany

**Keywords:** Unified european registry for inherited metabolic diseases, U-IMD, Inherited metabolic diseases, European infrastructure for rare diseases, ERDRI, European reference network for rare hereditary metabolic disorders, MetabERN

## Abstract

**Background:**

Following the broad application of new analytical methods, more and more pathophysiological processes in previously unknown diseases have been elucidated. The spectrum of clinical presentation of rare inherited metabolic diseases (IMDs) is broad and ranges from single organ involvement to multisystemic diseases. With the aim of overcoming the limited knowledge about the natural course, current diagnostic and therapeutic approaches, the project has established the first unified patient registry for IMDs that fully meets the requirements of the European Infrastructure for Rare Diseases (ERDRI).

**Results:**

In collaboration with the European Reference Network for Rare Hereditary Metabolic Disorders (MetabERN), the Unified European registry for Inherited Metabolic Diseases (U-IMD) was established to collect patient data as an observational, non-interventional natural history study. Following the recommendations of the ERDRI the U-IMD registry uses common data elements to define the IMDs, report the clinical phenotype, describe the biochemical markers and to capture the drug treatment. Until today, more than 1100 IMD patients have been registered.

**Conclusion:**

The U-IMD registry is the first observational, non-interventional patient registry that encompasses all known IMDs. Full semantic interoperability for other registries has been achieved, as demonstrated by the use of a minimum common core data set for equivalent description of metabolic patients in U-IMD and in the patient registry of the European Rare Kidney Disease Reference Network (ERKNet). In conclusion, the U-IMD registry will contribute to a better understanding of the long-term course of IMDs and improved patients care by understanding the natural disease course and by enabling an optimization of diagnostic and therapeutic strategies.

## Background

According to the Inborn Errors of Metabolism Knowledgebase (IEMbase, http://www.iembase.org), the most comprehensive online knowledge base for inherited metabolic diseases (IMDs), more than 1600 IMDs (as of December 2020) have been identified to date [6]. The numbers are increasing rapidly, since new analytical high through-put methods help to unravel the molecular and metabolic basis hitherto unknown diseases [5]. While, each IMD is a rare condition with estimated individual prevalence ranging from 0.1 to 15 in 100,000 newborns, taken together patients affected by IMDs are numerous with at least one in 500 newborns. Most IMDs manifest in the newborn period and infancy although increasing numbers of patients with adult onset forms of IMDs are being recognized. Depending on the underlying defect and its individual severity, the phenotypic spectrum of IMDs is broad ranging from involvement of single organ systems to multi-systemic disease [22]. Individuals with IMDs are frequently confronted with significant and often severe health problems resulting in high morbidity, reduced life expectancy, and poor quality of life [20].

Since IMDs are rare, experience of single expert centres is extremely limited and the evidence base of current diagnostic and therapeutic approaches is still low for the majority of IMDs [21]. As a consequence of this and in addition to an often-delayed diagnosis, significant inequalities for patients with IMDs exist. Causative therapies such as dietary treatment, substrate reduction therapy, cofactor treatment, enzyme replacement therapy, and transplantation (liver, kidney, bone marrow, hematopoietic stem cell therapy) are available for some IMDs. However, for most IMDs therapy is still symptomatic and does not sufficiently change the course of the disease [1].

Individuals with a rare disease have become a healthcare priority in the European Union (EU) Member States since 1999 as other causes of infant mortality such as most infectious disease are now treatable [19]. In this context European Reference Networks (ERNs) have been established as networks involving healthcare providers across Europe with the aim to tackle complex or rare diseases and conditions that require highly specialized treatment as well as a concentration of cross border knowledge and resources. For IMDs the European Reference Network for Rare Hereditary Metabolic Disorders (MetabERN) was established in 2017 (https://www.metab.ern-net.eu). It represents today 78 nationally certified Healthcare Providers from 23 European Member States and 44 patient organizations. MetabERN is thus the first comprehensive, pan-metabolic, pan-European, patient-oriented, clinical and research platform ever conceived worldwide.

In analogy, the European Rare Kidney Disease Reference Network (ERKNet) is seeking to improve standards of diagnosis and treatment for patients affected by rare and complex kidney diseases across Europe. ERKNet has been identified as a strategic partner for joint registry efforts such as the development of standards for minimal core datasets for the registries of both networks since patients with certain IMDs included in MetabERN also develop acute or chronic renal disease manifestations.

The aim of the presented project is to establish the first unified patient registry for Inherited Metabolic Diseases named “Unified European registry for Inherited Metabolic Diseases “(U-IMD) under the umbrella of MetabERN. Previously, successful networking activities and patient registries on a European or international level existed for only a limited number of IMDs. Examples are the EU-funded European Registry and Network for Intoxication type Metabolic Diseases (E-IMD: https://www.eimd-registry.org/; CHAFEA agreement no. 2010 12 01), the European Network and Registry for Homocystinurias and Methylation Defects (E-HOD: https://www.ehod-registry.org/; CHAFEA agreement no. 2012 12 02), and the patient registry of the International Working Group on Neurotransmitter Related Disorders (iNTD: https://www.intd-registry.org/). These projects have described in detail the natural history of more than 40 different IMDs and their disease variants, evaluated the impact of interventional and non-interventional parameters (including evaluation of newborn screening programmes for IMDs if available), and developed evidence-based care protocols being the basis for national guidelines and being translated into information brochures for patients, families and healthcare professionals [2–4, 7–13, 13, 14, 14–18, 23]. Thereby, the E-IMD, E-HOD and iNTD family of rare disease registries has confirmed that networking activities and the establishment of patient registries have a relevant impact for improving the health of patients with IMDs, and for harmonizing diagnostic algorithms, therapy and long-term follow-up and care on a European and international level.

However, existing patient registry projects described the natural history for only a few known IMDs. Furthermore, the registries are not fully conforming to the requirements of the European Rare Disease Registry Infrastructure (ERDRI), thus interoperability of records produced by these registries is not fully granted. A major next step would therefore be to expand the registry to all 1685 IMDs (as of December 2020) listed in the IEMbase and to upgrade already existing registries (including E-IMD, E-HOD, iNTD) to the ERDRI standard for rare disease registration. This approach improves the knowledge base, fosters networking on a European level among healthcare professionals and stakeholders, and lays the basis for future clinical trials and post-authorization surveillance measures of orphan drugs for all IMDs in Europe.

## Results

### Governance and structure of U-IMD

With the aim of establishing the first European registry covering all known IMDs, the beneficiaries of U-IMD successfully applied for the call HP-PJ-06-2016 of the EU Executive Agency for Consumer, Health, Agriculture and Food (CHAFEA). The prerequisite for a successful application to this call was that the new established registry fully complies with the guidelines of the European Union Committee of Experts on Rare Diseases (EUCERD) and the European Commission Expert Group on Rare Diseases (CEG-RD), while at the same time fully implementing the ERDRI standard for rare disease registries.

The U-IMD beneficiaries are members of MetabERN as well as members of multiple IMD networks and are from experienced metabolic expert centres in four EU countries (Czech Republic, Germany, Italy and Spain), covering approximately 45% of the EU population. By including MetabERN and the established IMD networks E-IMD, E-HOD and iNTD as collaborating stakeholders the project further extended the geographical coverage. U-IMD furthermore invited non-MetabERN health care providers to become part of the project. Becoming part of U-IMD is possible for all health care providers by receiving a positive vote for the U-IMD study protocol by the responsible body and by the accepting the U-IMD consortium agreement. Patient involvement is secured through the patient advocacy groups organized within MetabERN.

U-IMD is coordinated by Heidelberg University Hospital and consists of the three bodies: (1) Members Board, (2) Steering Group, and (3) Work Packages, according to the CHAEFEA requirements.

1. The Members Board comprises all members of MetabERN that signed the U-IMD letter of agreement. This is a direct measure for assuring that U-IMD is fashioned according the requirements of MetabERN and for guaranteeing managerial continuity after the end of the funding period. The Members Board has an advisory function and is the principle decision making and arbitration body of the project, deciding by simple majority. By opening the Members Board to all MetabERN members, patient involvement is secured through the patient advocacy groups organized within MetabERN enjoying all obligations and privileges of a board member. The Members Board meets once a year in person, preferably during the annual Society for the Study of Inborn Errors of Metabolism (SSIEM) conference or annual MetabERN Members meeting receiving direct feedback on the proceedings of the project from the Work Package Leaders. Otherwise it is the obligation of the Steering Group to keep the Members Board informed. Members that use the registry for data entry are responsible for ensuring that their ethical authorization to collect data in the U-IMD registry is up to date and are responsible for applying the national guidelines and directives concerning patient consent. Beyond the major research aims of this project as specified in the grant agreement, any member may make an additional sufficiently elaborated request to the Members board to use the network’s data, which is the subject to voting rules as described above.
2. The Steering Group consists of the Work Package Leaders, one official representative from each of the three existing IMD networks (E-IMD, E-HOD, and iNTD), the MetabERN coordinator, and a member and/or representative of a patient advocacy group. The leader of Work Package 1—Coordination is project leader and chair of the steering group. The Chair presides over the meetings of the Steering Group. The Steering Group coordinates and monitors the implementation of the project according to the milestones and deliverables laid out in the project plan. The Steering Group meets regularly and at each meeting Work Package Leaders are expected to provide a comprehensive progress report on new developments, opportunities and challenges encountered during their operational work creating transparency, accountability and early awareness of obstacles. If necessary the Steering Group takes prompt and adequate action to help in tackling challenges or to decide on the implementation of contingencies.
3. All specific tasks to be fulfilled to reach the project aims are thematically bundled in Work Packages (Coordination, Dissemination, Evaluation and Patient registry) forming the centres of the operational work within the project.
  - Work Package 1—Coordination (Centre for Child and Adolescent Medicine, Heidelberg University Hospital, Germany) is responsible for project management, including overall coordination of all scientific and non-scientific issues. It has to ensure that measures are implemented, milestones reached and deliverables achieved and is the central liaison for stakeholders seeking collaboration. Furthermore, it is responsible for the strategic planning of long-term sustainability.
  - Work Packages 2—Dissemination (General University Hospital Prague, Czech Republic) is responsible for the distribution of projects proceedings and results via various communication tools (including the website https://u-imd.org/) among all stakeholders, including other research networks, professional societies, clinicians and scientists, patient and parent organisations, policy makers, general public and industry.
  - Work Package 3—Evaluation (Bambino Gesù Children’s Hospital Rome, Italy) does a continuous evaluation of the project against indicators defined for the specific objectives within the given time frame and managing the process of preparing the evaluation report. The evaluation process is intended to promote learning from the project and ensure the achievement of intended impact. Indicators are reaching of defined milestones and deliverables, quality and quantity of data collected in the registries, completeness of geographical coverage, coverage of the MetabERN disease panel, acceptance of U-IMD within MetabERN and full implementation of the ERDRI standards.
  - Work Package 4—Patient registry (Centre for Child and Adolescent Medicine, Heidelberg University Hospital, Germany) will establish and maintain the Unified European registry for Inherited Metabolic Diseases (U-IMD), by building on other registry projects including E-IMD, E-HOD, and iNTD and fully implementing the ERDRI standards. It will make the existing iNTD registry interoperable with the new unified registry and develop a minimal core data set to be shared with the upgraded ERKNet registry.

### U-IMD patient registry: set-up

**Fig. 1:**
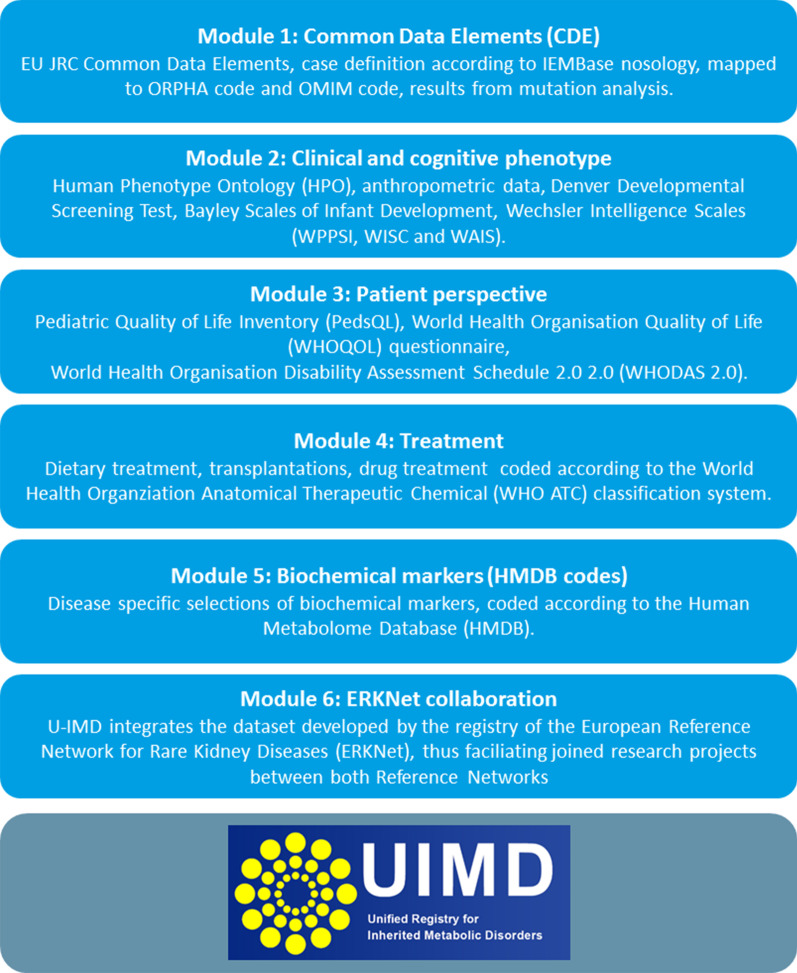
Overview on the structure of U-IMD and the corresponding modules

The newly established Unified European Registry for Inherited Metabolic Diseases is the core of the U-IMD project. This registry is implemented as an observational, non-interventional natural history study, requiring members to obtain an ethics committee approval for the U-IMD study protocol and informed consent forms according to local standards. Patients are followed prospectively using a set of common data elements that are collected at baseline and standardized follow-up visits collecting longitudinal data. The data model of the registry includes a broad spectrum of basic information (e.g. disease name, date and mode of diagnosis, genotype, sex) as well as detailed information on the clinical presentation, laboratory test results, treatment modalities (including dietary management), follow-up investigations, results of standardized psychological and developmental tests (e.g. BSID-III, WPPSI-IV, WISC-V, WAIS-IV), patient and parent questionnaires on quality of life as well as on the level of disability for adult patients (Fig. 1). The registry is web-based and accessible by password-protected user accounts via the internet using a secure encrypted connection.

The U-IMD registry follows the recommendations of ERDRI on rare disease patient registration. The key guiding principle of ERDRI is the usage of systematic and controlled vocabularies for coding diagnosis and describing phenotypic abnormalities as well as the usage of core data sets that are similar across multiple platforms. It hereby aims at increasing the interoperability of European rare disease registries and reducing the fragmentation of data sources (https://eu-rd-platform.jrc.ec.europa.eu/). The applied set of tools consists of four major elements:

- *Common Data Elements (CDE):* A set of variables and guiding principles that should be adhered by each rare disease registry in the EU, allowing to describe patients identically at least on the level of this core data set.
- *European Patient Identity Service (EUPID):* GDPR-compliant pseudonymization tool for patient registration, providing distinct pseudonyms for patients in different contexts while retaining a protected link between the different pseudonyms. The EUPID services acts as trusted third party supporting the creation of merged datasets.
- *European Directory of Registries (ERDRI.dor):* “Registry of registries” for European rare disease registries, searchable by up to 27 characteristics, like name of registry, disease panel, operating institution, etc.
- *Central Metadata Repository (ERDRI.mdr):* Collects the metadata of all data elements used by participating registries, like variable names, definition of variables, value domains, etc.

The U-IMD registry is designed in a modular fashion and integrates the ERDRI recommendations as follows (Fig. 1):

#### Common Data Elements (CDE)

The registry contains a module of common data elements that is uniform across all diseases. The module contains all data elements recommended by ERDRI and additional variables specific to the context of IMDs. Cases are defined using the nosology of the IEMbase which is mapped to ORPHA codes and OMIM gene codes (see Additional file 1: Table S1). Disease-causing mutations are recorded according to the systematics of the ClinVar, Human Gene Mutation Database (HGMD) and the Single Nucleotide Polymorphism database (dbSNP). Biochemical markers at diagnosis are collected according to the disease specific sets developed by the IEMbase which are mapped to the coding system of the Human Metabolome Database (HMDB; http://www.hmdb.ca/).

#### Clinical phenotype and patient perspective

The clinical phenotype is recorded using the structured and controlled vocabulary of the Human Phenotype Ontology (http://human-phenotype-ontology.github.io/), ensuring interoperability with other registries using the same vocabulary for phenotyping and comparability of phenotypes across all diseases in the registry.

#### Patient perspective

The registry also contains a module for adequately capturing the patient perspective that is uniform across all diseases. PedsQL (http://www.pedsql.org/) and WHOQOL-BREF questionnaires (http://www.who.int/mental_health/publications/whoqol/en/) can be filled in by the patients directly and have been translated and validated in multiple languages. Disability is measured by the World Health Organization Disability Assessment Schedule (WHODAS 2.0).

#### Biochemical phenotype

The evolving biochemical phenotype is recorded according to the disease specific sets of biochemical markers developed by the IEMbase which are mapped to the coding system of the Human Metabolome Database (HMDB; http://www.hmdb.ca/). The Human Metabolome Database is a freely available electronic database containing detailed information about small molecule metabolites found in the human body.

#### Treatment

The registry comprises also a module for capturing drug treatment that is uniform across all diseases. For this purpose, the World Health Organization Anatomical Therapeutic Chemical Classification (WHO ATC) classification system (https://www.whocc.no/atc_ddd_index/) is used representing an established, controlled and standardized vocabulary. WHO ATC codes drugs with a five-tiered classification system, from the treated organ system down to the respective chemical substance. Off-label or orphan drug usage is recorded as recommended by EUCERD. Some patients with IMDs require special diet and the most common dietary treatments have been added to the data model.

### U-IMD patient registry: current status of recruitment

**Fig. 2:**
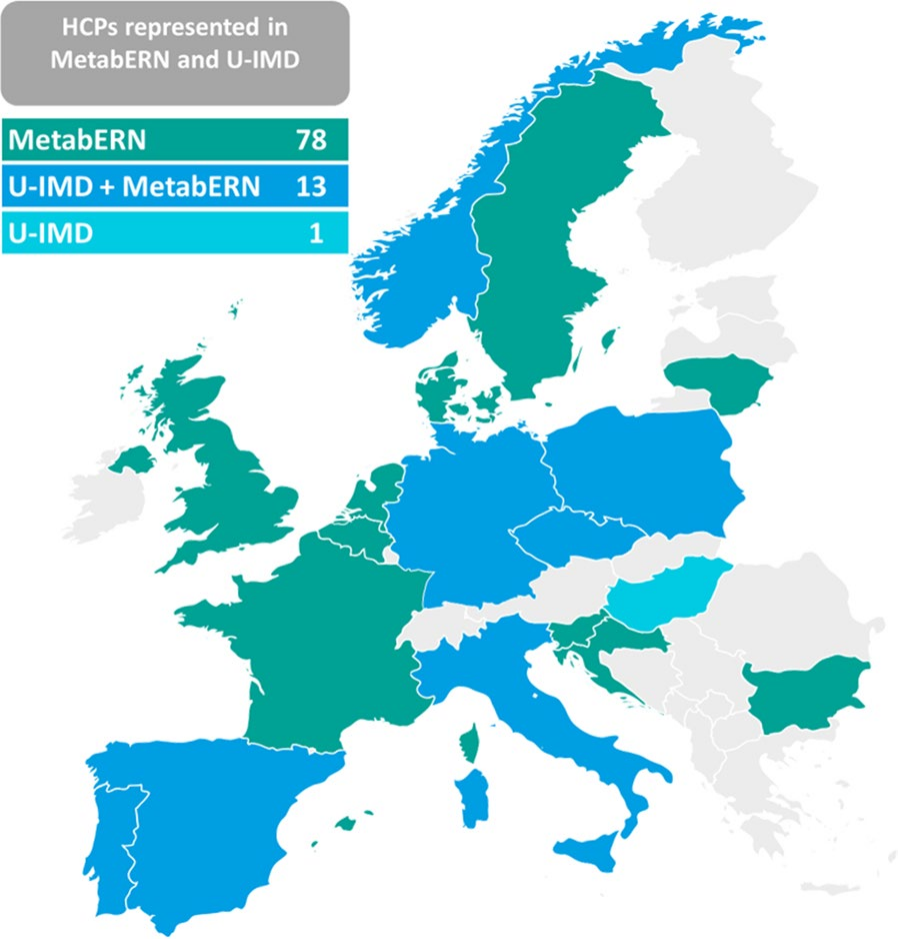
Geographical coverage of the U-IMD registry in Europe

The patient registry was successfully launched in February 2019, after being approved by the local ethics committee of the coordinating centre (i.e. University Hospital Heidelberg, application no. S-387/2018) on 5th July, 2018 and then approved by the ethics committees of clinical partners contributing to the registry. Fourteen MetabERN partners and one non-MetabERN partner from nine European countries [8 EU member states (MS)], have so far contributed to the registry (Fig. 2).

**Fig. 3:**
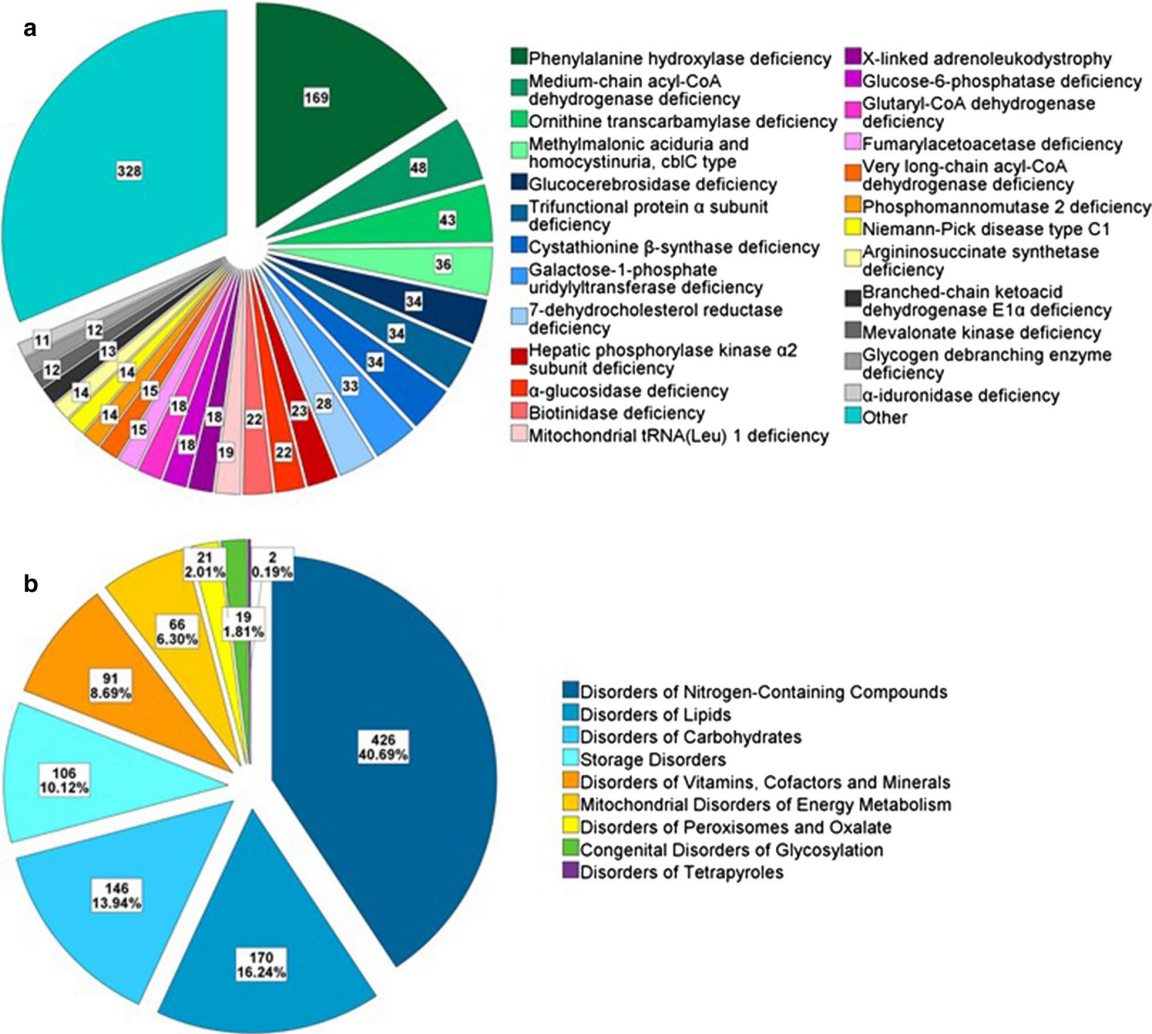
**a** The 25 most frequently recorded diseases in the registry. **b** Distribution of the individual disease groups in the patient register

By October 2020, 1193 individuals with a confirmed IMD from 13 different European centers have been registered. The distribution of the 25 most frequently documented IMDs in the registry is shown in Fig. 3. A detailed overview of all in the U-IMD registry documented IMDs, the corresponding number of patients as well as the IEM codes can be found in the Additional file 1: Tables S1 and S2.

#### Upgrade of existing IMD registries

To improve semantic interoperability and ERDRI conformity of all patient records coming from the various IMD registries, it is our strategy to upgrade existing IMD registries to the standard of data collection developed for U-IMD. In the framework of the project, the iNTD registry serves as a pilot for this adaptation process and was enhanced by the inclusion of the ERDRI CDEs, IEMbase nosology for case definition and HPO terms of description of the phenotype. Until today 34 iNTD patients records from the University Children’s Hospital Heidelberg have been updated using new functions.

#### Collaboration with ERKNet

To facilitate the collaboration between MetabERN and ERKNet, the ERNs developed a shared minimal core data set. The ERKNet registry has been upgraded and U-IMD programmed using this core dataset, ensuring that metabolic nephropathies are described alike in both registries. As of June 2020, 14 patients have been registered in U-IMD using the shared core dataset.

## Discussion

Patient registries are increasingly recognized as a powerful tool for assessing disease course, understanding variations in diagnosis, treatment and outcomes, and investigating factors affecting prognosis and quality of life. On the other hand, patient registries often cover only individual diseases or groups of diseases, have different standards of data quality or are subject to commercial interests. The diversity of the registry landscape with its inconsistent structures leads to a great time burden for the health care providers with sometimes duplicated efforts.

The Unified European registry for Inherited Metabolic Diseases is the first observational, non-interventional patient registry established by academia that encompasses all 1600 + IMDs (as of December 2020). Eighteen months after the official initiation the registry comprises already data on more than 1100 IMD patients from 14 MetabERN partners and one non-MetabERN partner in 9 European countries.

By implementing the ERDRI requirements in the U-IMD registry full semantic interoperability for other IMD registries has been achieved. Using the iNTD registry, an existing registry for individuals with neurotransmitter-associated IMDs started in 2014, as a pilot we established a template to upgrade existing IMD registries improving their semantic interoperability and complying with ERDRI. Similarly, the cooperation between U-IMD and ERKNet was successfully documented by using a core dataset.

Noteworthy, U-IMD does not aim to replace existing IMD registries. U-IMD will offer knowhow transfer to those consortia which are willing to upgrade existing IMD registries by implementation of interoperable data modules developed by U-IMD. By this, existing IMD registries can enhance semantic interoperability of patient records and reach EUCERD conformity, facilitating cross-site data exchange and analysis in a General Data Protection Regulation (GDPR)-compliant framework. In U-IMD, in analogy to other existing IMD registries (E-IMD, E-HOD, and iNTD), the health care provider (HCP) is and shall remain the owner of any protected or unprotected intellectual property rights.

It is expected that the continuation of this positive development will lead to a better understanding of the long-term course of the disease and the outcome as well as a clear identification of disease variants for IMD. Furthermore, it will enable an evaluation of genotype/phenotype correlations, and an optimization of diagnostic and therapeutic strategies, which in the long run will not only shorten the diagnostic odyssey of patients but also improve the long-term clinical outcomes of affected patients.

### Ethical approval

The ethical review process required for the assessment of longitudinal patient data needs to be regarded as a major drawback for the activation process of individual sites and, as a consequence, for patient recruitment. For data sharing within or between ERNs a consent form has been developed (https://cpms-training.ern-net.eu/cms/media/consent/EN_InformedConsent.pdf). By signing the form, patients and parents agree to share data collected at their hospital with other ERN healthcare professionals. Such data may include medical images or reports and laboratory reports and would provide therefore offer an ideal basis for data collection within a patient registry. However, the ERN Patient Consent Form for Data Sharing has found to be insufficient for the use with U-IMD registry (and analogous projects), since the evaluation of the patient registry has to be regarded as research and therefore has to follow the EU General Data Protection Regulation (EU-GDPR, https://ec.europa.eu/info/law/law-topic/data-protection_en, Enforced since 25.05.2018). Any research study that aims to evaluate and publish data requires a separate ethic approval and informed consent process.

Since there are no agreed rules for low or even no risk non-interventional or observational research studies on European level, the ethical review process is laborious and bureaucratic for U-IMD centres in some countries. For future projects under the umbrella of a European Reference Networks it would therefore be of great benefit to harmonize regulations for the ethical review process. In addition, a standardized consent form that would allow ERN healthcare professionals to make available and share patient data stored in healthcare records would ensure data extraction, exchange, and cross-site analysis between databases, registries or research projects in a GDPR-protected framework.

### Platform for clinical research and trial

The data gathered in the U-IMD registry is pseudonymized by using the EUPID service. Members can only access their own patient data and always retain full data ownership. Usage of the entire U-IMD dataset is governed by the U-IMD Consortium, granting members equal rights in initiating and deciding on mutual projects. Any member may make specific and sufficiently elaborated requests to the Members Board to use the network’s data for clinical research. In addition, for describing the natural course of IMDs or other related clinical research, the U-IMD registry can serve as an excellent platform for the screening of patient cohorts and the organization of clinical research studies and trials. In the context of the recent increase in innovative and partially individualized treatments, such patient registries can also provide detailed safety and efficacy monitoring after drug approval. It hereby contributes to a facilitated approval of orphan drugs.

### Outlook and participation

U-IMD has established a European registry for all known IMDs. To structure the pathway of data collection for such a broad spectrum of diseases and a large number of affected individuals the members of the seven subnetworks of MetabERN will identify disease-specific projects on regular basis, such as natural history studies. These selected projects and diseases will be given priority for data collection for the forthcoming year. This strategy helps to stepwise increase the number of diseases and affected individuals in the U-IMD registry.

By strictly following ERDRI standards the U-IMD registry is well suited to cooperate with existing or newly established resources in the field of rare diseases, such as databases or platforms. Through semantic interoperability, it also provides a basis for machine learning or the use of other advanced digital tools including artificial intelligence AI algorithms. Hereby, even for rare diseases a significant shortening of the path to diagnosis could be achieved in future. U-IMD is collaborating in the context of ERDRI, Solve-RD and EJP-RD to make its datasets a findable asset that could be merged with compatible datasets, using the EUPID and RD-NEXUS services. Being the official registry of MetabERN it is also open to all European and international health care providers, following individuals with IMDs. Supporting information and material can be obtained by contacting U-IMD via the project or registry website.

## Conclusion

The Unified European registry for Inherited Metabolic Diseases (U-IMD) is the first observational, non-interventional patient registry that encompasses all known inherited metabolic diseases. Interoperability with other rare disease registries has been ensured by fulfilment of the requirements of the European Rare Disease Registry Infrastructure (ERDRI).

## Supplementary Information

**Additional file 1: Table S1.** List of IEM codes collected in the U-IMD patient registry. **Table S2**. Detailed list of diseases collected in the U-IMD patient registry with the corresponding number of patients.

## Data Availability

The datasets used and/or analysed during the current study are available from the corresponding author on reasonable request.

## Abbreviations

U-IMD: Unified European registry for Inherited Metabolic Diseases
IMD: Inherited metabolic disease
ERDRI: European Rare Disease Registry Infrastructure
MetabERN: European Reference Network for Rare Hereditary Metabolic Disorders;
RD: Rare disease
ERKNet: European Rare Kidney Disease Reference Network
IEMbase: Inborn Errors of Metabolism Knowledgebase
EU: European Union
E-IMD: European Registry and Network for Intoxication type Metabolic Diseases
E-HOD: European Network and Registry for Homocystinurias and Methylation Defects
iNTD: International Working Group on Neurotransmitter Related Disorders
CHAFEA: EU Executive Agency for Consumer, Health, Agriculture and Food
SSIEM: Society for the Study of Inborn Errors of Metabolism
WHO ATC: World Health Organization Anatomical Therapeutic Chemical Classification
HPO: Human Phenotype Ontology
EU-GDPR: EU General Data Protection Regulation
HMDB: The Human Metabolome Database
EUCERD: European Union Committee of Experts on Rare Diseases
CEG-RD: European Commission Expert Group on Rare Diseases
WHOQOL-BREF: World Health Organization Quality of Life
WHODAS: World Health Organization Disability Assessment Schedule
PedsQL: Pediatric Quality of Life Inventory
