# Supplementary material for: U-IMD: the first Unified European registry for inherited metabolic diseases
Source: Orphanet J Rare Dis. 2021 Feb 18;16:95. doi: 10.1186/s13023-021-01726-3 (PMC7893973; doi:10.1186/s13023-021-01726-3)
Supplement: Supplementary file 1 — Additional file 1: Table S1. List of IEM codes collected in the U-IMD patient registry. Table S2. Detailed list of diseases collected in the U-IMD patient registry with the corresponding number of patients. [file 13023_2021_1726_MOESM1_ESM.docx]

**Additional files:**

Additional table 1:

| IEM0007 | Phosphoribosylpyrophosphate synthetaseÂ superactivity |
| --- | --- |
| IEM0016 | Xanthine oxidase deficiency |
| IEM0017 | Hypoxanthine guanine phosphoribosyltransferase deficiency |
| IEM0018 | Adenine phosphoribosyltransferase deficiency |
| IEM0068 | Cystinuria type A |
| IEM0069 | Cystinuria type B |
| IEM0070 | Lysinuric protein intolerance |
| IEM0126 | Methylmalonyl-CoA epimerase deficiency / |
| IEM0127 | Methylmalonic aciduria due to methylmalonyl-CoA mutase deficiency |
| IEM0128 | Acyl-CoAÂ synthetase family member 3 deficiency |
| IEM0218 | Methylmalonic aciduria, cblA type |
| IEM0219 | Methylmalonic aciduria, cblB type |
| IEM0269 | Vitamin D 24-hydroxylase deficiency |
| IEM0278 | Molybdenum cofactor sulfurase deficiency |
| IEM0370 | Glucose-6-phosphatase deficiency |
| IEM0387 | Lactate dehydrogenase A deficiency |
| IEM0410 | S-adenosylmethionine carrier deficiency |
| IEM0503 | Mitochondrial RNA import protein deficiency |
| IEM0504 | Ribonuclease P 5' tRNA processing enzyme deficiency |
| IEM0505 | Ribonuclease Z 3' tRNA processing enzyme deficiency |
| IEM0508 | Mitochondrial methionyl-tRNA formyltransferase deficiency |
| IEM0509 | tRNA 5-taurinomethyluridine modifier deficiency |
| IEM0510 | tRNA 5-carboxymethylaminomethyl transferase deficiency |
| IEM0513 | tRNA methyltransferase 5 deficiency |
| IEM0516 | Mitochondrial ribosomal large subunit 3 deficiency |
| IEM0517 | Mitochondrial ribosomal large subunit 44 deficiency |
| IEM0519 | Mitochondrial ribosomal small subunit 16 deficiency |
| IEM0520 | Mitochondrial ribosomal small subunit 22 deficiency |
| IEM0521 | Mitochondrial ribosomal small subunit 34 deficiency |
| IEM0524 | RMND1 deficiency |
| IEM0525 | Mitochondrial elongation factor G1 deficiency |
| IEM0527 | Mitochondrial elongation factor Ts deficiency |
| IEM0528 | Mitochondrial elongation factor Tu deficiency |
| IEM0529 | C12orf65 release factor deficiency |
| IEM0552 | Mitochondrial alanyl-tRNA synthetase deficiency |
| IEM0554 | Mitochondrial asparaginyl-tRNA synthetase deficiency |
| IEM0556 | Mitochondrial cysteinyl-tRNA synthetase deficiency |
| IEM0557 | Mitochondrial glutamyl-tRNA synthetase deficiency |
| IEM0562 | Mitochondrial phenylalanyl-tRNA synthetase deficiency |
| IEM0565 | Mitochondrial valyl-tRNA synthetase deficiency |
| IEM0593 | Mitochondrial intermediate peptidase deficiency |
| IEM0609 | Sideroflexin 4 deficiency |
| IEM0610 | AIFM1 deficiency |
| IEM0613 | C1q binding protein deficiency |
| IEM0734 | Lecithin cholesterol acyltransferase deficiency |
| IEM0844 | α-Galactosidase A deficiency |
| IEM0873 | Cystinosin deficiency |
| IEM0903 | Alanine-glyoxylate aminotransferase deficiency |
| IEM0905 | Glyoxylate reductase/hydroxypyruvate reductase deficiency |
| IEM0906 | 4-hydroxy-2-oxoglutarate aldolase 1 deficiency |

Additional table 2:

| **Group** | **Sub-Group** | **Disease** | **Count** |
| --- | --- | --- | --- |
| Disorders of Nitrogen-Containing Compounds | Disorders of purine metabolism | Hypoxanthine guanine phosphoribosyltransferase deficiency | **2** |
|  | Disorders of creatine metabolism | Creatine transporter deficiency | **2** |
|  | Disorders of choline metabolism | Flavin monooxygenase 3 deficiency | **1** |
|  | Disorders of ammonia detoxification | N-acetylglutamate synthase deficiency | **1** |
|  |  | Carbamoylphosphate synthetase I deficiency | **3** |
|  |  | Ornithine transcarbamylase deficiency | **43** |
|  |  | Argininosuccinate synthetase deficiency | **14** |
|  |  | Argininosuccinate lyase deficiency | **9** |
|  |  | Mitochondrial ornithine transporter deficiency | **4** |
|  |  | Carbonic anhydrase VA deficiency | **1** |
|  | Disorders of amino acid transport | Cystinuria type A | **4** |
|  |  | Cystinuria type B | **1** |
|  |  | Lysinuric protein intolerance | **2** |
|  | Aminoacylase deficiencies | Aspartoacylase deficiency | **1** |
|  |  | Aminoacylase 1 deficiency | **1** |
|  | Disorders of monoamine metabolism | Tyrosine hydroxylase deficiency | **1** |
|  |  | Aromatic L-amino acid decarboxylase deficiency | **1** |
|  | Disorders of phenylalanine and tetrahydrobiopterin metabolism | Phenylalanine hydroxylase deficiency | **169** |
|  |  | Autosomal recessive GTP cyclohydrolase 1 deficiency | **1** |
|  |  | Autosomal dominant GTP cyclohydrolase 1 deficiency | **1** |
|  |  | 6-pyruvoyl-tetrahydropterin synthase deficiency | **4** |
|  |  | Dihydropteridine reductase deficiency | **1** |
|  | Disorders of tyrosine metabolism | Homogentisic acid oxidase deficiency | **2** |
|  |  | Fumarylacetoacetase deficiency | **15** |
|  | Disorders of sulfur amino acid and sulfide metabolism | Methionine adenosyltransferase I/III deficiency | **1** |
|  |  | S-adenosylhomocysteine hydrolase deficiency | **2** |
|  |  | Adenosine kinase deficiency | **1** |
|  |  | Cystathionine β-synthase deficiency | **34** |
|  |  | Cystathionine γ-lyase deficiency | **2** |
|  |  | Mitochondrial sulfur dioxygenase deficiency | **2** |
|  | Disorders of branched-chain amino acid metabolism | Branched-chain ketoacid dehydrogenase E1α deficiency | **13** |
|  |  | Branched-chain ketoacid dehydrogenase E1β deficiency | **9** |
|  |  | Dihydrolipoyl transacylase deficiency | **1** |
|  |  | Branched-chain ketoacid dehydrogenase kinase deficiency | **1** |
|  |  | Isovaleryl-CoA dehydrogenase deficiency | **8** |
|  |  | 2-Methylbutyryl-CoA dehydrogenase deficiency | **2** |
|  |  | 3-Methylcrotonyl-CoA carboxylase 1 deficiency | **1** |
|  |  | 3-Methylcrotonyl-CoA carboxylase 2 deficiency | **1** |
|  |  | 3-methylglutaconyl-CoA hydratase deficiency | **1** |
|  |  | Mitochondrial short-chain enoyl-CoA hydratase 1 deficiency | **2** |
|  |  | HSD10 disease | **1** |
|  |  | 3-Hydroxy-3-methylglutaryl-CoA lyase deficiency | **3** |
|  |  | Propionic acidemia due to propionyl-CoA carboxylase α subunit deficiency | **8** |
|  |  | Propionic acidemia due to propionyl-CoA carboxylase β subunit deficiency | **6** |
|  |  | Methylmalonic aciduria due to methylmalonyl-CoA mutase deficiency | **10** |
|  | Disorders of lysine metabolism | α-aminoadipic semialdehyde dehydrogenase deficiency | **3** |
|  |  | Glutaryl-CoA dehydrogenase deficiency | **18** |
|  | Disorders of glutamate metabolism | Glutamate dehydrogenase superactivity | **5** |
|  |  | Ionotropic glutamate receptor NMDA type subunit 2A dysregulation | **1** |
|  |  | Ionotropic glutamate receptor NMDA type subunit 2B dysregulation | **4** |
|  | Disorders of glycine metabolism | Glycine encephalopathy due to glycine decarboxylase deficiency | **1** |
|  |  | Glycine encephalopathy due to aminomethyltransferase deficiency | **1** |
| Disorders of Vitamins, Cofactors and Minerals | Disorders of cobalamin metabolism | Hereditary intrinsic factor deficiency | **1** |
|  |  | Transcobalamin receptor deficiency | **1** |
|  |  | Methylmalonic aciduria and homocystinuria, cblJ type | **1** |
|  |  | Methylmalonic aciduria and homocystinuria, cblC type | **36** |
|  |  | cblD disease | **1** |
|  |  | Methionine synthase reductase deficiency | **1** |
|  |  | Methionine synthase deficiency | **5** |
|  |  | Methylmalonic aciduria, cblA type | **2** |
|  |  | Methylmalonic aciduria, cblB type | **3** |
|  |  | Methylmalonic aciduria and homocystinuria, cblX type | **1** |
|  | Disorders of folate metabolism | 5,10-methylenetetrahydrofolate reductase deficiency | **4** |
|  | Disorders of biotin metabolism | Biotinidase deficiency | **22** |
|  |  | Holocarboxylase synthetase deficiency | **3** |
|  | Disorders of thiamine metabolism | Mitochondrial thiamine pyrophosphate transporter deficiency | **1** |
|  | Disorders of riboflavin metabolism | Riboflavin transporter 3 deficiency | **1** |
|  |  | Electron transfer flavoprotein α subunit deficiency | **2** |
|  |  | Electron transfer flavoprotein dehydrogenase deficiency | **3** |
|  | Disorders of pyridoxine metabolism | Tissue-nonspecific alkaline phosphatase deficiency | **2** |
|  | Disorders of molybdenum metabolism | Cyclic pyranopterin monophosphate synthase deficiency | **1** |
| Disorders of Carbohydrates | Disorders of carbohydrate transport and absorption | Blood-brain barrier glucose transporter 1 deficiency | **6** |
|  |  | Congenital sucrase-isomaltase deficiency | **1** |
|  | Disorders of galactose metabolism | Galactose-1-phosphate uridylyltransferase deficiency | **33** |
|  |  | Galactokinase deficiency | **2** |
|  | Disorders of fructose metabolism | Aldolase B deficiency | **5** |
|  | Disorders of the pentose phosphate pathway and polyol metabolism | Glucose-6-phosphate dehydrogenase deficiency | **2** |
|  | Disorders of insulin secretion and signaling | ATP-sensitive potassium channel regulatory subunit deficiency | **2** |
|  |  | Uncoupling protein 2 deficiency | **1** |
|  | Glycogen storage diseases | Hepatic glycogen synthase deficiency | **3** |
|  |  | Glucose-6-phosphate transporter deficiency | **7** |
|  |  | α-glucosidase deficiency | **22** |
|  |  | Glycogen debranching enzyme deficiency | **12** |
|  |  | Glycogen branching enzyme deficiency | **1** |
|  |  | Hepatic phosphorylase kinase α2 subunit deficiency | **23** |
|  |  | Phosphorylase kinase β subunit deficiency | **2** |
|  | Disorders of gluconeogenesis | Glucose-6-phosphatase deficiency | **18** |
|  |  | Fructose-1,6-bisphosphatase deficiency | **3** |
|  |  | Pyruvate carboxylase deficiency | **1** |
|  | Disorders of glycolysis | Glucokinase superactivity | **1** |
|  |  | Muscle phosphofructokinase deficiency | **1** |
| Mitochondrial Disorders of Energy Metabolism | Disorders of pyruvate metabolism | Pyruvate dehydrogenase E1-α deficiency | **3** |
|  | Disorders of metabolite repair | L-2-hydroxyglutarate dehydrogenase deficiency | **1** |
|  | Disorders of mitochondrial carriers | Cytosolic glycerol-3-phosphate dehydrogenase deficiency | **3** |
|  | Disorders of complex I subunits | NADH dehydrogenase flavoprotein 1 deficiency | **1** |
|  |  | NADH dehydrogenase iron-sulfur protein 8 deficiency | **1** |
|  |  | NADH dehydrogenase iron-sulfur protein 4 deficiency | **1** |
|  |  | NADH dehydrogenase core subunit 1 deficiency | **3** |
|  |  | NADH dehydrogenase core subunit 4 deficiency | **4** |
|  |  | NADH dehydrogenase core subunit 5 deficiency | **4** |
|  | Disorders of complex I assembly | NADH dehydrogenase α subcomplex assembly factor 6 deficiency | **1** |
|  | Disorders of complex II subunits | Succinate dehydrogenase subunit A deficiency | **2** |
|  | Disorders of complex III assembly | LYRM7 deficiency | **1** |
|  | Disorders of complex IV assembly and ancillary proteins | SURF1 deficiency | **1** |
|  |  | APOPT1 deficiency | **1** |
|  | Disorders of complex V subunits | Mitochondrial ATP synthase F0 subunit 6 deficiency | **2** |
|  | Disorders of complex V assembly | Transmembrane protein 70 deficiency | **1** |
|  | Disorders of mitochondrial DNA depletion, multiple deletion, or intergenomic communication | Mitochondrial deoxyguanosine kinase deficiency | **1** |
|  | Disorders of mitochondrial tRNA | Mitochondrial tRNA(Leu) 1 deficiency | **19** |
|  |  | Mitochondrial tRNA(Lys) deficiency | **3** |
|  | Disorders of mitochondrial tRNA incorporation and recycling | Mitochondrial aspartyl-tRNA synthetase deficiency | **1** |
|  |  | Mitochondrial glutamyl-tRNA synthetase deficiency | **1** |
|  |  | Mitochondrial phenylalanyl-tRNA synthetase deficiency | **1** |
|  |  | Mitochondrial tryptophanyl-tRNA synthetase deficiency | **1** |
|  | Disorders of mitochondrial fusion | OPA1 deficiency | **5** |
|  | Disorders of mitochondrial phospholipid metabolism | Tafazin deficiency | **3** |
|  | Primary CoQ10 deficiencies | COQ4 deficiency | **1** |
| Disorders of Lipids | Disorders of carnitine metabolism | Primary carnitine deficiency | **3** |
|  |  | Carnitine palmitoyltransferase 1A deficiency | **1** |
|  |  | Carnitine palmitoyltransferase 2 deficiency | **2** |
|  |  | Carnitine-acylcarnitine translocase deficiency | **2** |
|  | Disorders of fatty acid oxidation and transport | Short-chain acyl-CoA dehydrogenase deficiency | **2** |
|  |  | Medium-chain acyl-CoA dehydrogenase deficiency | **48** |
|  |  | Very long-chain acyl-CoA dehydrogenase deficiency | **15** |
|  |  | Trifunctional protein α subunit deficiency | **34** |
|  |  | Trifunctional protein β subunit deficiency | **4** |
|  |  | TANGO2 deficiency | **3** |
|  | Disorders of ketone body metabolism | Mitochondrial acetoacetyl-CoA thiolase deficiency | **4** |
|  | Disorder of fatty aldehyde metabolism | Fatty aldehyde dehydrogenase deficiency | **1** |
|  | Disorders of cytoplasmic triglyceride metabolism | Lysophosphatidic acid acyltransferase deficiency | **1** |
|  |  | CGI-58 deficiency | **1** |
|  | Disorders of phosphoinositide metabolism | Phosphatidylinositol 3,4,5-trisphosphate 5-phosphatase deficiency | **1** |
|  | Disorders of lipoprotein metabolism | Hypercholesterolemia due to ligand-defective apo B | **1** |
|  |  | Apolipoprotein B deficiency | **1** |
|  |  | Sitosterolemia due to ABCG5 deficiency | **1** |
|  |  | Lipoprotein lipase deficiency | **2** |
|  | Disorders of cholesterol biosynthesis | Mevalonate kinase deficiency | **12** |
|  |  | 24-dehydrocholesterol reductase deficiency | **1** |
|  |  | 7-dehydrocholesterol reductase deficiency | **28** |
|  | Disorders of bile acid synthesis | Sterol 27-hydroxylase deficiency | **2** |
| Disorders of Tetrapyroles | Disorders of heme metabolism | Ferrochelatase deficiency | **1** |
|  | Disorders of bilirubin metabolism and biliary transport | UDP-glucuronosyltransferase A1 deficiency | **1** |
| Storage Disorders | Disorders of autophagy | EPG5 deficiency | **1** |
|  | Neuronal ceroid lipofuscinosis | Tripeptidyl-peptidase 1 deficiency | **2** |
|  | Sphingolipidoses | Glucocerebrosidase deficiency | **34** |
|  |  | β-galactosidase deficiency, GM1 gangliosidosis phenotype | **4** |
|  |  | β-hexosaminidase α-subunit deficiency | **2** |
|  |  | β-hexosaminidase β-subunit deficiency | **1** |
|  |  | β-galactosylceramidase deficiency | **1** |
|  |  | Arylsulfatase A deficiency | **2** |
|  |  | α-Galactosidase A deficiency | **6** |
|  | Mucolipidoses | UDP-N-acetylglucosamine-1-phosphotransferase γ subunit deficiency | **1** |
|  | Mucopolysaccharidoses | α-iduronidase deficiency | **11** |
|  |  | Iduronate sulfatase deficiency | **4** |
|  |  | Heparan N-sulfatase deficiency | **4** |
|  |  | N-acetylglucosaminidase deficiency | **2** |
|  |  | N-acetylgalactosamine 6-sulfatase deficiency | **5** |
|  |  | N-acetylgalactosamine 4-sulfatase deficiency | **2** |
|  | Disorders of lysosomal cholesterol metabolism | Niemann-Pick disease type C1 | **14** |
|  |  | Lysosomal acid lipase deficiency | **8** |
|  | Disorders of lysosomal transport or sorting | Cystinosin deficiency | **2** |
| Disorders of Peroxisomes and Oxalate | Disorders of plasmalogen synthesis | Glycerone 3-phosphate acyltransferase deficiency | **1** |
|  | Disorders of peroxisomal ß-oxidation | X-linked adrenoleukodystrophy | **18** |
|  | Disorder of peroxisomal ß-oxidation | Phytanoyl-CoA hydroxylase deficiency | **1** |
|  | Disorders of peroxisomal biogenesis | Peroxin 14 deficiency | **1** |
| Congenital Disorders of Glycosylation | Disorders of N-linked glycosylation | Phosphomannomutase 2 deficiency | **14** |
|  |  | X-linked recessive UDP-N-acetylglucosamine transferase catalytic subunit deficiency | **1** |
|  |  | Lipid-linked oligosaccharide flippase deficiency | **1** |
|  |  | ALG3 α-1,3-mannosyltransferase deficiency | **1** |
|  |  | ALG6 α-1,3-glucosyltransferase deficiency | **1** |
|  | Disorders of monosaccharide synthesis and interconversion | Phosphoglucomutase 1 deficiency | **1** |
| **Total** | | | **1047** |
